# Predictive value of bile acids as metabolite biomarkers for gallstones: A protocol of systematic review and meta-analysis

**DOI:** 10.1371/journal.pone.0284138

**Published:** 2023-04-19

**Authors:** Xu Han, Juan Wang, Yingnan Wu, Hao Gu, Ning Zhao, Xing Liao, Miao Jiang

**Affiliations:** 1 Institute of Basic Research in Clinical Medicine, China Academy of Chinese Medical Sciences, Beijing, China; 2 Department of Traditional Chinese Medicine, Inner Mongolia People’s Hospital, Hohhot, China; Universidad Tecnica de Manabi, ECUADOR

## Abstract

**Background:**

Plenty of studies have focused on the bile acids profile in gallstones. The objective of our systematic review is to provide a comprehensive summary about bile acids profiles in gallstones and analyzes the difference between gallstones and control group in diverse samples, determining the characteristic bile acids as the metabolite biomarkers for predicting gallstone.

**Methods:**

EMBASE, the Cochrane Library, PubMed, Web of Science, Wanfang databases, China National Knowledge Infrastructure (CNKI), VIP Information Resource Integration Service Platform (CQVIP), and China Biology Medicine Disc (SinoMed) will be searched with the keywords of gallstones and metabolomics. The screening process will be performed strictly according to inclusion and exclusion criteria. The CONSORT checklist and the Newcastle-Ottawa Scale (NOS) will assess the risk of bias for randomized controlled trials and observational studies, respectively. The qualitative review will be conducted to summarize the bile acids profile in gallstones. The concentrations of bile acids in both case group and control group will be the primary outcomes to perform the meta-analyses.

**Expected results:**

Our systematic review will find the characteristic bile acids as the candidate metabolite biomarkers which equipped potential value to predict gallstones.

**Conclusion:**

Expanding the current knowledge on the physiopathology of gallstones and identifying novel predictive biomarkers can help to facilitate the detection and management of gallstones. Consequently, we expect this protocol to be a reasonable method to filtrate candidate differential bile acids which have potential value to predict gallstones.

**PROSPERO registration number:**

CRD42022339649.

## Introduction

Gallstone disease is one of the most common gastrointestinal diseases with a high prevalence rate reaching 20% in developed countries and 10% in China [1, 2]. Gallstones can be classified as cholesterol gallstones, pigment gallstones (black and brown) and mixed gallstones according to the calculus composition and appearance [3]. Cholesterol cholelithiasis is the main type, which are composed of a higher percentage of cholesterol and calcium salts of bilirubin and phosphates [4]. Due to the huge change of lifestyle and economic development, the prevalence rate of gallstones keeps increasing worldwide year by year, consequently the risks of the severe complications such as cholecystitis and pancreatitis rise as well [5, 6]. In addition, gallstone is reported to be the most important risk factor for gallbladder cancer (GBC) [7], which leads to a huge financial burden. The newly study reported that the total expenditure of biliary tract diseases in the United States reached 16.9 billion dollars in 2021 [8].

The typical symptoms of cholesterol cholelithiasis include intense abdominal pain, fever, nausea, vomiting and jaundice [3]. Current mainstream therapies include pain relief with analgesics, oral litholysis with ursodeoxycholic acid (UDCA), and routine open or laparoscopic cholecystectomy [4], with the therapeutic goals aiming at controlling symptoms, avoiding recurrence, and preventing complications.

Once lithogenesis happens and the gallstone reaches a certain size, cholecystectomy is the gold standard therapy for symptomatic gallstone patients with biliary pain or complications [9]. However, nonspecific postsurgical gastrointestinal symptoms like persistent abdominal pain and dyspepsia occur in up to 10% of cases [10]. Moreover, multifarious post operative complications, for example injuries of bile ducts, bile leaks, bleeding, intestinal injuries, and infection, may occur. All these result in the multiplication of patient pain and the significant rise in healthcare costs. One review claimed that surgery was the wrong solution in the long run, and it suggest that much more emphasis should be given to the prevention of gallstones [11]. Conservative treatment is also popular in clinic, for example, UDCA is prescribed to dissolve the stone, yet the applied conditions are strict, and the risk of gallstone recurrence is as high as 30–43% of patients within 3 to 5 years [12, 13]. Thus, an optimal strategy for gallstone treatment may be avoiding the gallstones formation in advance, compared to treating symptomatic gallstones [11].

To achieve the goal of preventive treatment, the vital step is to discover the predictive indicators of gallstone occurrence before lithogenesis. Bile acids have specific physiological functions and chemical properties and are promising biomarkers for predicting stone formation. Bile acids are synthesized in hepatocytes and secreted into the intestinal tract, as an important component of bile stored in the gallbladder [14]. The confusion of bile acids homeostasis plays a key role in the process of gallstone formation, because the slight transformation of bile acids motives the huge effect for gallstones development. On the other hand, bile acids have a substantial function in digestion, absorption, and metabolism. As a signaling molecule, bile acids impact various receptors like Farnesoid X receptor (FXR) and G-protein coupled receptor (TGR5), leading to change not only bile acids metabolism but also glucose homeostasis, lipid and lipoprotein metabolism, energy expenditure, intestinal motility, bacterial growth, inflammation, and the liver-gut axis [15, 16], and all these molecules and pathways are involved in the lithogenesis. Therefore, increasingly researchers have focused on the underlying value of bile acids in predicting, diagnosing, and treating various diseases, especially hepatic and gall diseases.

However, as a group of substances, the changes of bile acids profile are complex. Metabolomics is the suitable platform to determine the overall changes of bile acids profile qualitatively and quantitatively, which makes it possible to characterize the state of the body with bile acids profile [17]. This technology that is revolutionized by advances in liquid chromatography-mass spectrometry past decade has been successfully applied to help the researchers discover key metabolites and their associations with diseases [18].

Therefore, an increasing number of metabolomics studies have focused on the bile acids profile in gallstones, and demonstrate that the levels of some bile acids are significantly associated with the development of gallstones [19–21]. However, there is still lack of a consistent and comprehensive conclusion and the biological samples they detect are diverge. Thus, a systematic review is necessitated to be performed based on the published literature. This protocol of systematic review aims to state the specific method of the systematic review about how to provide a comprehensive summary of bile acids profiles in gallstones and determine the characteristic bile acids as the potential metabolite biomarkers for predicting gallstones by analyzing the difference between gallstones and control group in diverse samples. The systematic summary will provide a clearer direction for future research, and the quality evaluation will serve as the reference for criteria of quality assessment to standardize and promote the quality of related publications. The discovery of bile acid biomarkers can identify risk populations before gallstones develop and subsequently advance the time of intervention, which will reduce patient suffering and avoiding cholecystectomy. The findings should offer more solid and reliable evidence and foundation for the exploration of bile acid metabolism mechanism during the formation of gallstones, which will be the basis for the fresh and deep research in this field.

## Methods

Our systematic review will be performed and reported by the PRISMA 2020 statement [22]. We registered this protocol in the International Prospective Register of Systematic Reviews (PROSPERO) database, number CRD42022339649.

### Ethics and dissemination

This systematic review is based on published researches, so there is no ethical approval required. We intend to disseminate our findings in a peer-reviewed journal.

### Review question

Through reviewing the published studies, we aim to summarize the bile acids profile of gallstones by metabolomics, to find the characteristic bile acids between gallstones and control group, exploring the value of bile acids as biomarkers for predicting gallstones.

### Eligibility criteria

#### Type of population

Inclusion criteria: Adult patients diagnosed with gallstones. Exclusion criteria: 1) Patients with acute simple cholecystitis, acute suppurative cholecystitis, acute gangrenous cholecystitis, acute obstructive suppurative cholecystitis, gallbladder perforation complicated with diffuse peritonitis; 2) Patients who are receiving bile acids drugs like UDCA.

#### Type of intervention

Metabolomics was applied to measure and analyze biological samples, such as blood, urine, feces, and bile, obtained from all participants.

#### Type of comparator

The control group are individuals without gallstones.

#### Type of outcomes

The variation trend or concentration of bile acids between gallstones and control group.

#### Type of studies

The randomized controlled trial and observational study will be included.

In addition, duplicate records, insufficient information, and the study for which the full text is not available will be excluded.

### Search strategy

Records will be searched during the period from June to August 2022 through the following databases: EMBASE, the Cochrane Library, PubMed, Web of Science, Wanfang databases, China National Knowledge Infrastructure (CNKI), VIP Information Resource Integration Service Platform (CQVIP), and China Biology Medicine Disc (SinoMed) with the following keywords: (Gallstones OR Cholelithiasis OR Cholecystolithiasis OR Choledocholithiasis) AND (Metabolomics OR Metabolome OR Metabolic fingerprinting). Medical subject headings (Mesh) terms will be used for the PubMed, Cochrane Library and SinoMed search, and Emtree explode terms for the Embase. The language will be restricted to English in PubMed, Embase, Web of Science and Cochrane Library, and Chinese in Wanfang databases, CNKI, CQVIP and SinoMed. No time restriction will be applied. Human clinical studies will be confined.

### Study selection

**Fig 1:**
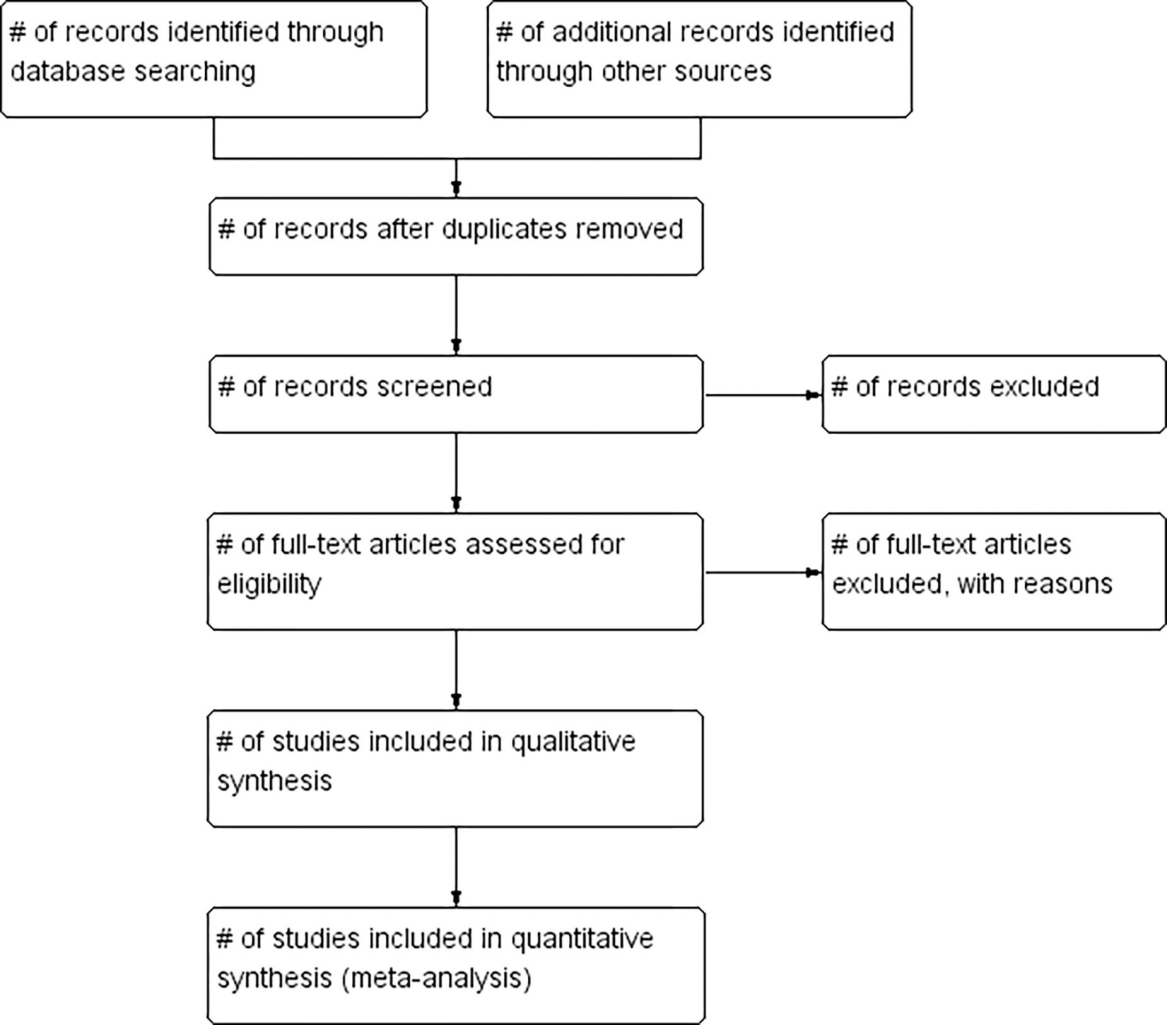
Flow chart of study selection.

All identified records will be moved to Endnote X9, then duplicate records will be deleted. Two independent researchers (JW and YNW) will screen the studies by title and abstract respectively, then eligible records will be downloaded full text for further screening. Any disagreements will be discussed with the third researcher (MJ) until the team reached a consensus. A senior researcher (XL) will supervise the whole process. The flow chart is shown in the Fig 1.

### Risk of bias assessment

Two independent researchers (JW and YNW) will use the CONSORT checklist [23] and Newcastle-Ottawa Scale (NOS) (http://www.ohri.ca/programs/clinical_epidemiology/oxford.asp) to assess the risk of bias for randomized controlled trial and observational study, respectively. The CONSORT 2010 checklist was used to assess the reporting level of the included randomized trial. The specific rules for scoring each item/subitem were made according to CONSORT Explanation and Elaboration (E&E) document. The NOS is a tool that estimates the quality of observational study by a “star system” with three aspects included selection, comparability and exposure [24].

### Data collection

Data of included studies will be extracted and the following items will be contain: the name of the first author; year of publication; country of origin; language; type of study design; the sample size; age of each group; diagnostic criteria of patients; type of gallstones (cholesterol gallstones, pigment gallstones or mixed gallstones); the biological samples for examining (blood, urine, feces or bile); metabolomics technique; the variation trend and concentration of bile acids both in gallstones group and control group; evidence of independent validation cohort (if available). The data will be derived from the article and the article’s additional file. If the data is not available from published documents, corresponding author will be contacted for raw data. All obtained data will be recorded and managed in Microsoft Excel.

### Data synthesis

The qualitative review will be conducted to describe and summarize the bile acids profile of gallstones form identified studies by counting the statistically significant trend of differential bile acids between gallstones and control group. Then, the quantitative review will be performed by meta-analyses to quantify the degree of variation of bile acids, which concentrations will be the primary outcomes and the weighted mean difference (WMD) and confidence intervals 95% (95% CI) will be calculated. Notably, concentrations with different units will be standardized to the appropriate units before meta-analyses, and the median with quartile or interquartile range (IQR) will be converted into mean with standard deviation (SD) by the statistical method and the formula, respectively. The statistical method combined a way that tested the skewness and a new piecewise function based on the size of sample [25–27]. The formula was provided by the Cochrane handbook for systematic reviews of interventions and shown in Table 1. The random-effects model will be selected because it incorporates both within- and between-study components of variance. I^2^ statistic will be used to measure heterogeneity, which over 30% will be considered as substantial heterogeneity. Sensitivity analyses will be performed by removing the high risk of studies and by sequential omitting each study. Subgroup analysis will be performed according to study design, biological samples, and analytic technique. Funnel plots and the Egger’s test will assess publication bias when feasible (10 or more studies) [28, 29]. All the data synthesis will be performed by R software (Version 3.6.2) with meta package.

**Table 1:** Formula for converting median and interquartile range (IQR) into mean and standard deviation (SD).

Item	Formula
Mean	Mean = Median
SD	SD = IQR/1.35

### Expected results

We anticipate that the systematic review will provide a comprehensive summary of bile acids profiles in gallstones and find the characteristic bile acids as the candidate metabolite biomarkers which equipped potential value to predict gallstones.

## Discussion

The composition of bile contains cholesterol, bile acids, phospholipids, bilirubin, fatty acids, vitamins and minerals [30]. Cholesterol supersaturation in gallbladder bile is the major contributing factor to the formation of gallstones, which caused by imbalance of cholesterol, bile acids and phospholipid [31]. However, the exactly machines are still vague. Duo to the key role of bile acids in gallstones formation, plenty studies have further explored the relationship between bile acids and gallstones by metabolomics [32, 33]. A Swedish study showed that the shortage of bile acids was a major reason why bile was supersaturated with cholesterol in gallstones patients [34]. While secondary bile acids were higher in patients with gallstones compared with healthy controls [35], and higher overall concentrations of fecal bile acids with lower microbial diversity had been found in gallstones [36]. A prospective cohort study proved that the disorder of bile acids metabolism had already occurred at the beginning of the disease [37]. Thus, it is necessary to comprehensively summarize the changes of different species of bile acids in gallstones.

Separately, cholesterol cholelithiasis as well as had relationship with metabolic abnormalities included insulin resistance, expansion of visceral adiposity, overweight, obesity, type 2 diabetes, and the metabolic syndrome [4]. Bile acids as biological signaling molecules regulated metabolic response by inspiring dedicated receptors FXR and TGR5, being associated with metabolic disorders [38]. The previous study demonstrated that the repression of FXR in mice reduces cholesterol solubility in bile by decreasing the expression of CYP7A1 and hence hepatic bile salt synthesis [39]. Therefore, an intensive study of the changes in bile acids and the subsequently altered physiological functions is meaningful for investigating the mechanism of gallstone formation.

### Strengths and limitations

Recognizing the critical importance of bile acids in the pathogenesis of gallstones. A systematic review is essential to provide a comprehensive summary of bile acids profiles in gallstones and determine the characteristic bile acids as the potential metabolite biomarkers for predicting gallstones. This protocol is the reasonable and elaborate method to achieve this objective. It is notable that the protocol is designed with a special and comprehensive analysis method based on the bile acids property, which will include and analyze a variety of biological samples containing bile acids, such as blood, feces, and bile. The concrete systematic review will be strictly in accordance with the methodology of the protocol. The limitations of the study protocol are that the results of meta-analysis could be highly heterogeneous, which is caused by inter-study variability in population and methodology, and this may lead to lower quality of evidence. The protocol will minimize the heterogeneity and enhance the reliability of the results through subgroup analysis and sensitivity analysis.

## Conclusion

Expanding the current knowledge on the physiopathology of gallstones and identifying novel predictive biomarkers can help to facilitate the detection and management of gallstones. Consequently, we expect this protocol to be a reasonable method to filtrate candidate differential bile acids which have potential value to predict gallstones.

## Supporting information

S1 Checklist Reporting checklist for protocol of a systematic review and meta analysis. (DOCX)

## Abbreviations

CNKI: China National Knowledge Infrastructure
CQVIP: VIP Information Resource Integration Service Platform
SinoMed: China Biology Medicine Disc
NOS: Newcastle-Ottawa Scale
GBC: Gallbladder Cancer
UDCA: Ursodeoxycholic acid
FXR: Farnesoid X receptor
TGR5: G-protein coupled receptor
Mesh: Medical subject headings
WMD: Weighted mean difference
95% CI: Confidence intervals 95%
